# Daily Physical Activities and Sports in Adult Survivors of Childhood Cancer and Healthy Controls: A Population-Based Questionnaire Survey

**DOI:** 10.1371/journal.pone.0034930

**Published:** 2012-04-10

**Authors:** Corina S. Rueegg, Nicolas X. von der Weid, Cornelia E. Rebholz, Gisela Michel, Marcel Zwahlen, Michael Grotzer, Claudia E. Kuehni, for the Swiss Paediatric Oncology Group (SPOG)

**Affiliations:** 1 Swiss Childhood Cancer Registry, Institute of Social and Preventive Medicine, University of Bern, Bern, Switzerland; 2 Paediatric Hematology-Oncology Unit, Centre Hospitalier Universitaire Vaudois, Lausanne, Switzerland; 3 Department of Oncology, University Children's Hospital Zurich, Zurich, Switzerland; Universidad Europea de Madrid, Spain

## Abstract

**Background:**

Healthy lifestyle including sufficient physical activity may mitigate or prevent adverse long-term effects of childhood cancer. We described daily physical activities and sports in childhood cancer survivors and controls, and assessed determinants of both activity patterns.

**Methodology/Principal Findings:**

The Swiss Childhood Cancer Survivor Study is a questionnaire survey including all children diagnosed with cancer 1976–2003 at age 0–15 years, registered in the Swiss Childhood Cancer Registry, who survived ≥5years and reached adulthood (≥20years). Controls came from the population-based Swiss Health Survey. We compared the two populations and determined risk factors for both outcomes in separate multivariable logistic regression models. The sample included 1058 survivors and 5593 controls (response rates 78% and 66%). Sufficient daily physical activities were reported by 52% (n = 521) of survivors and 37% (n = 2069) of controls (p<0.001). In contrast, 62% (n = 640) of survivors and 65% (n = 3635) of controls reported engaging in sports (p = 0.067). Risk factors for insufficient daily activities in both populations were: older age (OR for ≥35years: 1.5, 95CI 1.2–2.0), female gender (OR 1.6, 95CI 1.3–1.9), French/Italian Speaking (OR 1.4, 95CI 1.1–1.7), and higher education (OR for university education: 2.0, 95CI 1.5–2.6). Risk factors for no sports were: being a survivor (OR 1.3, 95CI 1.1–1.6), older age (OR for ≥35years: 1.4, 95CI 1.1–1.8), migration background (OR 1.5, 95CI 1.3–1.8), French/Italian speaking (OR 1.4, 95CI 1.2–1.7), lower education (OR for compulsory schooling only: 1.6, 95CI 1.2–2.2), being married (OR 1.7, 95CI 1.5–2.0), having children (OR 1.3, 95CI 1.4–1.9), obesity (OR 2.4, 95CI 1.7–3.3), and smoking (OR 1.7, 95CI 1.5–2.1). Type of diagnosis was only associated with sports.

**Conclusions/Significance:**

Physical activity levels in survivors were lower than recommended, but comparable to controls and mainly determined by socio-demographic and cultural factors. Strategies to improve physical activity levels could be similar as for the general population.

## Introduction

Survival rates of childhood cancer reached 80% [1], [2], resulting in a growing population of adult childhood cancer survivors. This has shifted to some extent the focus of childhood cancer treatment and its follow-up from saving the patient's life to preserving long-term health. Due to genetic predisposition or therapy, childhood cancer survivors are at increased risk for developing late effects such as pain, fatigue, obesity, diabetes, cardiovascular diseases, osteoporosis, cognitive problems, neuro-musculoskeletal complications, low functional capacity or late mortality [3], [4], [5], [6], [7], [8]. An active lifestyle has been shown to mitigate or prevent these problems in childhood cancer survivors [9], [10], [11], [12], [13], [14], [15], [16], survivors of adult cancer [17], [18], [19] and animal studies [20].

Two main types of physical activities have been described: 1) moderate or vigorous activities of daily living such as cleaning, gardening, walking, cycling or climbing stairs, labeled here as “daily activities”; and 2) targeted sports practiced on a regular basis such as soccer, jogging or gymnastics, labeled in this paper as “sports”. For health benefits, international guidelines recommend at least 30 minutes of moderate activities on at least 5 days per week or 20 minutes of vigorous activities on at least 3 days per week [21]. Increasing time of physical activity was shown to provide even greater health benefits [21], [22].

Despite its importance for maintenance of general health, relatively little is known about activity patterns of adult survivors of childhood cancer [14], [23]. Studies from the USA showed varying proportions of active survivors (between 36% and 80%) [23], [24], [25], [26], [27], [28]. Two of them compared results to healthy controls and distinguished between different types of physical activities [24], [25]. Four studies assessed determinants of physical activity; their results were inconsistent [23], [24], [25], [26], [29]. For Europe, we only found one report on physical activity in European childhood cancer survivors. This was a single-center study conducted in Finland based on a small sample of 21 survivors and controls [30].

We analyzed data from the nationwide Swiss Childhood Cancer Survivor Study and population-based controls from the Swiss Health Survey to compare daily activities and sports between the two populations, and to determine risk factors for insufficient daily activities and lack of sports.

## Materials and Methods

### Ethics statement

Ethics approval was provided through the general cancer registry permission of the Swiss Childhood Cancer Registry (The Swiss Federal Commission of Experts for Professional Secrecy in Medical Research) and a non obstat statement was obtained from the ethics committee of the canton of Bern, stating that no additional informed consent was necessary for the Swiss Childhood Cancer Survivor Study. All information regarding individuals from the Swiss Childhood Cancer Survivor Study as well as the Swiss Health Survey was made anonymous to investigators prior to analysis.

### The Swiss Childhood Cancer Survivor Study (SCCSS)

The SCCSS is a population-based long-term follow-up study of all childhood cancer patients registered in the Swiss Childhood Cancer Registry (SCCR), diagnosed between 1976 and 2003 at an age of 0–15 years, who survived at least 5 years [31]. The SCCR includes all children and adolescents in Switzerland diagnosed with leukemia, lymphoma, central nervous system (CNS) tumors, malignant solid tumors or Langerhans cell histiocytosis before age16 years [32], [33].

In 2007–2009, eligible study participants (N = 1686) were traced with an extensive address search procedure. They received an information letter from their former pediatric oncology clinic, followed by a questionnaire with a pre-paid return envelope. Non-responders received a second questionnaire four weeks later and were then reminded by phone. Letters and questionnaires were provided in three national languages: German, French and Italian (http://www.childhoodcancerregistry.ch/sccss). The SCCSS used an extensive questionnaire derived from the US and British childhood cancer survivor studies [34], [35]. Questions on health behaviors and socio-demographic measures were added from the Swiss Health Survey 2007 [36] and the Swiss Census 2000 [37], to allow comparisons with the Swiss general population. The main domains of the questionnaire were: quality of life, somatic health, fertility, current medication and health service utilization, psychological distress, health behaviors, and socio-economic information.

### The Swiss Health Survey (SHS)

The SHS is a periodical (every 5 years), representative telephone survey to describe the state of health and related questions of the Swiss resident population. We used data from the 2007 survey. Detailed information on the study procedure and questionnaire are provided on the homepage of the Swiss Statistics (http://www.bfs.admin.ch/bfs/portal/de/index/infothek/erhebungen__quellen/blank/blank/ess/03.html). Briefly, a random sample was obtained by stratified selection on two stages: in the first stage, households were randomly selected from each canton with oversampling in 14 cantons. In the second stage one person was randomly selected in each household. The sampling list consisted of 30'179 addresses of households. Thereof 1847 addresses were not valid. Of the remaining 28'332 households, 6185 households were unavailable and 3414 persons refused to participate, resulting in a sample of 18'760 interviewed persons (response rate 66%) [36]. The interview of participants was done by a computer assisted phone interview (CATI). The questionnaire included close to 400 questions grouped in 69 domains, which comprised questions on socio-demographic and socio-economic status, on health status, health related behaviours and health related attitudes as well as on the utilisation of health services. For this study, we only included SHS respondents aged 20–40 years according to the age range of survivors (n = 5593).

### Assessment of physical activity

In both populations, we assessed two different types of physical activities using similarly worded standardized questions (**Figure S1**) [36], [38], [39].

1. “Daily activities”: first, all participants were asked on how many days per week they engage in any type of activities that make them sweat (vigorous activities). Survivors were also asked how many minutes per day they engage in such activities. Then, participants were asked how many days per week and minutes per day they engage in activities that cause some increase in breathing (moderate activities). Examples were given for both types of activities in the questionnaires. People were then classified as “active” (vs. “inactive”) if they engaged in moderate activities for ≥30 minutes on ≥5 days a week or in vigorous activities on ≥3 days a week, according to the international physical activity recommendations of the Centers of Disease Control and Prevention (CDC) [21].
2. “Sporting activities”: participants were asked whether they engage in any gymnastics, fitness training or sports; how intensely they practice these sports; and how many hours per week. People were then classified as doing “sports” (vs. “no sports”) if they engaged in a targeted gym or sport at least somewhat intense and at least one hour per week. Survivors were also asked to list the types of sport they were doing.

### Information assessed by questionnaire

Explanatory variables assessed by questionnaire for both populations were: age at survey, gender, language region of Switzerland (German vs. French/Italian), civil status (married vs. single/divorced/other), having children, smoking status, migration background, education, and body mass index (BMI). Education was classified into four categories according to the Swiss Census: compulsory schooling only (≤9 years), vocational training (10–13 years), upper secondary education (higher vocational training or college), and university degree [37], [40]. Participants were classified as having a migration background if they were not Swiss citizens since birth, were not born in Switzerland, or at least one of the parents was not Swiss citizen. BMI was classified as underweight (BMI<18 kg/m^2^), normal weight (≥18–24.9 kg/m^2^), overweight (≥25–29.9 kg/m^2^), and obese (≥30 kg/m^2^) [41]. For survivors, self-reported late effects from cancer were assessed and coded into somatic or psychological late effects.

### Information extracted from the Swiss Childhood Cancer Registry (SCCR)

Prospectively collected medical information on diagnosis and treatment of survivors was extracted from the SCCR: age at diagnosis, cancer diagnosis, treatments, and time since diagnosis. Treatment was classified as surgery only, chemotherapy with or without surgery, radiotherapy with or without chemotherapy or surgery, and bone marrow transplantation. Diagnosis was classified according to the International Classification of Childhood Cancer - 3^rd^ Edition [42].

### Statistical Analysis

This analysis included all participants of the Swiss Childhood Cancer Survivor Study and the Swiss Health Survey aged 20 to 40 years at the time of survey. The Swiss Health Survey, our control population, included more migrants, French and Italian speaking, and older persons. On the combined data set we used multivariable logistic regression with being a control as outcome to compute appropriate weights to assure that the weighted marginal distribution of the control population for age, gender, language region, and migration background was identical to that in survivors (**Table 1**). The weight for the cancer survivors was set to one. We applied these weights in all analyses.

**Table 1:** Characteristics of the study population (Swiss Childhood Cancer Survivor Study) and the control population (Swiss Health Survey).

	Survivos (n = 1058)		Controls^a^ (n = 5593)		
Characteristics	n	%^b^	n	%	p^c^
**Current age (years)**					
≤24.9	417	39	2237	40	
25–29.9	304	29	1678	30	
30–34.9	188	18	1007	18	
≥35	149	14	671	12	na^d^
**Gender**					
Male	562	53	3020	54	
Female	496	47	2573	46	na^d^
**Migration background**					
No	798	75	4195	75	
Yes	260	25	1398	25	na^d^
**Language region of Switzerland**					
German speaking	815	77	4307	77	
French speaking	214	20	1118	20	
Italian speaking	29	3	168	3	na^d^
**Education**					
Compulsory schooling	93	9	336	6	
Vocational training	506	49	3635	65	
Upper secondary education	361	35	839	15	
University education	72	7	783	14	<0.001
**Civil status**					
Single, divorced, other	867	83	4139	74	
Married	177	17	1454	26	<0.001
**Children**					
No	920	87	2573	46	
Yes	138	13	3020	54	<0.001
**BMI categories (kg/m^2^)**					
Underweight (<18)	41	4	112	2	
Normal weight (≥18/<25)	698	68	4139	74	
Overweight (≥25/<30)	215	21	1063	19	
Obese (≥30)	72	7	279	5	0.001
**Smoking**					
Current non-smoker	792	76	3412	61	
Current smoker	250	24	2181	39	<0.001
**Age at diagnosis (years)**					
0–4.9	338	32			
5–9.9	279	26			
≥10	441	42			
**Time since diagnosis (years)**					
5–9.9	93	9			
10–19.9	471	45			
20–29.9	427	40			
≥30	67	6			
**Treatment**					
Surgery only	98	9			
Chemotherapy^e^	491	47			
Radiotherapy^f^	378	36			
Bone marrow transplantation	87	8			
**Diagnosis (ICCC-3 main groups)**					
I Leukemias	394	37			
II Lymphomas	219	21			
III CNS tumors	121	11			
IV Neuroblastomas	36	3			
V Retinoblastomas	21	2			
VI Renal tumors	60	6			
VII Hepatic tumors	7	1			
VIII Bone tumors	54	5			
IX Soft tissue sarcomas	58	6			
X Germ cell tumors	28	3			
Langerhans cell histiocytosis	44	4			
Other^g^	13	1			
**Self-reported late effects**					
No late effects	647	64			
Somatic late effects	259	26			
Psychological late effects	104	10			

Abbreviations: BMI, Body Mass Index; CNS, Central Nervous System; ICCC-3, International Classification of Childhood Cancer - Third Edition; na, not applicable.

a weighted proportions and numbers of the Swiss normal population according to the marginal distribution in survivors on: age, gender, language region, and nationality.

b percentages are based upon available data for each variable.

c p-value calculated from chi-square statistics.

d populations are weighted on these variables to make them comparable.

e chemotherapy may include surgery.

f radiotherapy may include surgery or chemotherapy.

g other malignant epithelial neoplasm, malignant melanomas and other or unspecified malignant neoplasm.

First, we used a combined dataset of survivors and controls to compare frequencies of outcomes and predictors using two-sample tests for proportions on Wald statistics. Means were compared using two-group comparison t-tests. We then fitted univariable and multivariable logistic regression models to determine associations of survivorship and other risk factors with the outcomes daily activities and sports. Variables that were significantly associated (p<0.05) with either of the two outcomes in the univariable model were included into the multivariable model. Wald tests were used to calculate global p-values. We used interaction tests to determine whether risk factors differed between the two populations, with a p<0.01 considered as statistically significant.

Second, we investigated associations between clinical factors (type of diagnosis and treatments) with the two types of physical activity, using the dataset of survivors only. P-values<0.05 were considered as statistically significant.

Analyses were performed with Stata, version 11.0 (Stata Corporation, Austin, Texas).

## Results

### Characteristics of the study population

**Figure 1:**
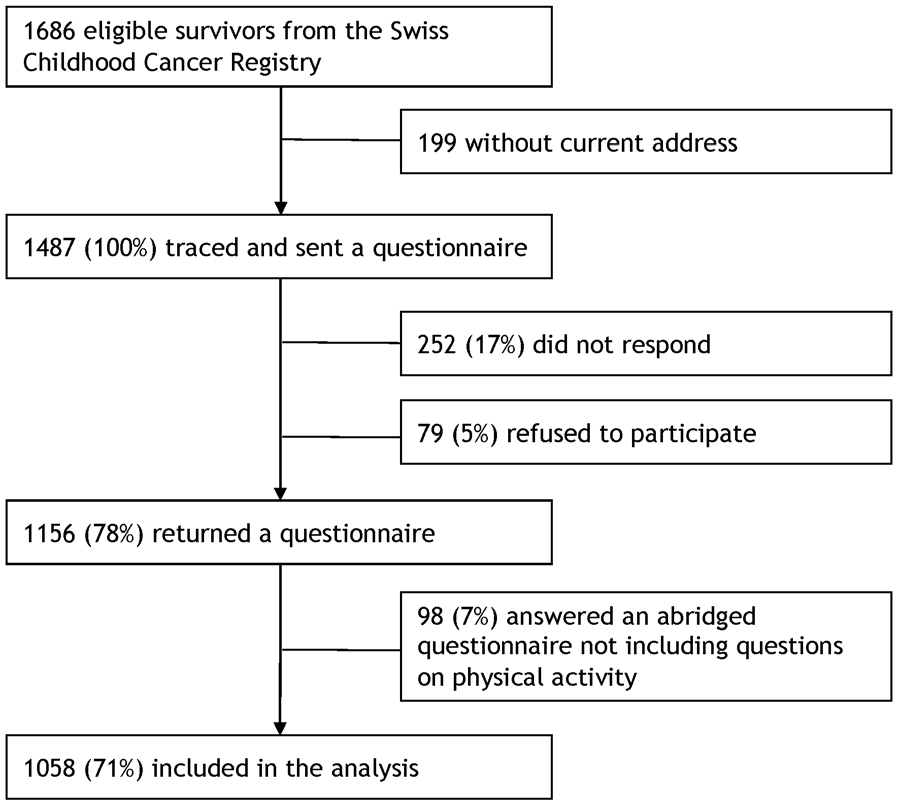
Participants of the Swiss Childhood Cancer Survivor Study. Flow diagram of our study population starting from those eligible in the Swiss Childhood Cancer Registry to those included in the analysis.

Addresses could be traced for 1487 of 1686 eligible survivors (**Figure 1**). Of those traced, 1156 (78%) returned a questionnaire, 1058 (71%) the full-length questionnaire and 98 (7%) an abbreviated version without questions on physical activity. Participants (n = 1058), in comparison to non-participants (n = 628), were more often female (47% vs. 38%) (p = <0.001), less often French (20% vs. 29%) or Italian (3% vs. 4%) speaking (p<0.001), had had more often leukemia (37% vs. 29%) (p = 0.008), and chemotherapy (47% vs. 44%) or bone marrow transplantation (8% vs. 3%) (p<0.001) (**Table S1**). They did not differ by current age, age at diagnosis, and time since diagnosis.

Compared to controls (**Table 1**), more survivors had compulsory schooling only (9% vs. 6%) or upper secondary education (35% vs. 15%), and fewer had vocational training (49% vs. 65%) or a university degree (7% vs. 14%; p<0.001). Fewer survivors were married (17% vs. 26%), had children (13% vs. 54%), or were current smoker (24% vs. 39%; all p<0.001). Mean BMI did not differ between the groups, but more survivors were underweight, overweight or obese (p = 0.001).

Most survivors had suffered from leukemia (37%), lymphoma (21%) or a CNS tumor (11%), 36% had been treated by radiotherapy and 8% with bone marrow transplant (**Table 1**). Mean age at diagnosis was 8.3 years (SD 4.8 years) and mean time elapsed since diagnosis 19.5 years (SD 6.6 years). About one third reported to suffer from late effects of their cancer disease.

### Daily activities and sports in survivors and controls

**Figure 2:**
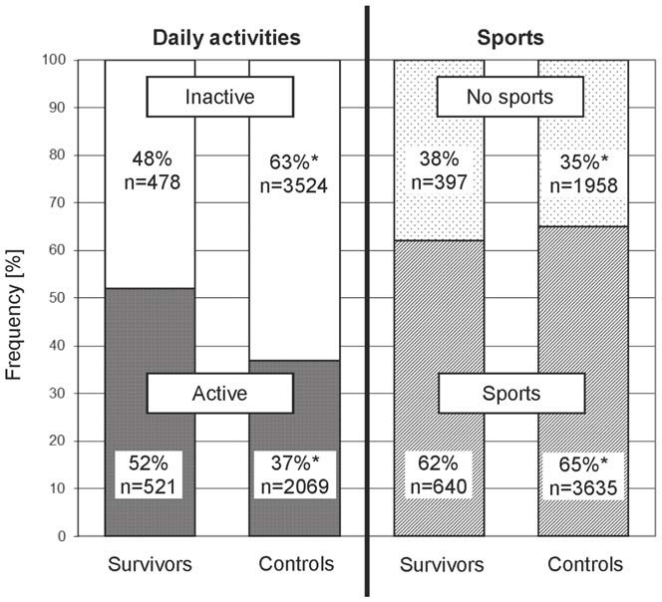
Proportion of survivors and controls reporting daily activities and sports. Proportions of persons 1) meeting (active) or not meeting (inactive) healthy recommendations for daily activities, and 2) participating (sports) or not participating (no sports) in sporting activities, for childhood cancer survivors compared to the weighted proportions of healthy controls (p<0.001 for daily activities, and p = 0.067 for sports, comparing survivors and controls). * Weighted proportions and numbers for controls: adjusted for age, gender, language region, and migration background.

Sufficient daily activities were reported by 52% (n = 521) of survivors and 37% (n = 2069) of controls (p<0.001, **Figure 2**). Sports participation was reported by 62% (n = 640) of survivors and 65% (n = 3635) of controls (p = 0.067). Survivors and controls had a similar proportion (21%) of completely inactive individuals, who neither engaged in sufficient daily activities nor in sports (data not shown). Survivors engaged in all typical types of sports, namely fitness training (reported by n = 385), outdoor activities (n = 170), ball games (n = 108), water sports (n = 97), rebounding ball games (n = 77), winter sports (n = 65), and others (n = 67; data not shown).

### Determinants of daily activities

Determinants of insufficient daily activities in a univariable logistic regression were: female sex (OR 1.6, 95CI 1.4–1.8), older age (OR for ≥35 years: 1.5, 95CI 1.2–1.9), higher education (OR for university degree: 2.3, 95CI 1.8–3.0), being married (OR 1.3, 95CI 1.1–1.6), having children (OR 1.3, 95CI 1.1–1.5), living in the French or Italian speaking part of Switzerland (OR 1.4, 95CI 1.2–1.6) and being underweight (OR 1.9, 95CI 1.2–3.1) (**Table S2**). Not associated were: migration background and smoking. Adjusting for all these variables, fewer survivors than controls reported insufficient daily activities (OR 0.5, 95CI 0.4–0.6) (**Table 2**). With the exception of civil status and having children all other variables remained associated in the adjusted model. Associations between risk factors and outcomes were similar for survivors and healthy peers. All p values for interaction were >0.01 apart from language region (p<0.001) and BMI (p = 0.006). Only French and Italian speaking survivors but not controls were at risk for insufficient daily activities. BMI was only associated in controls but not in survivors.

**Table 2:** Risk factors for inactivity and no sports from two multivariable regression models^a^ (combined dataset including childhood cancer survivors and controls).

	Daily activities						Sports					
	Inactive	Multivariable regression					No sports	Multivariable regression				
	%^b^	OR	95% CI			p^c^	%^b^	OR	95% CI			p^c^
**Population**												
Controls	63	1					36	1				
Survivors	48	0.48	0.41	-	0.58	<0.001	38	1.29	1.08	-	1.55	0.005
**Current age**												
0–24.9 years	50	1					35	1				
25–29.9 years	58	1.28	1.04	-	1.60		35	1.09	0.87	-	1.35	
30–34.9 years	58	1.39	1.09	-	1.78		40	1.12	0.87	-	1.44	
≥35 years	60	1.52	1.16	-	2.00	0.01	43	1.15	0.87	-	1.51	0.746
**Gender**												
Male	50	1					35	1				
Female	61	1.57	1.33	-	1.87	<0.001	39	1.18	0.99	-	1.39	0.06
**Migration background**												
No	54	1					34	1				
Yes	57	1.06	0.88	-	1.29	0.529	45	1.34	1.11	-	1.61	0.002
**Language region**												
German speaking	53	1					35	1				
French/Italian speaking	61	1.37	1.13	-	1.65	0.001	44	1.47	1.22	-	1.77	<0.001
**Education**												
Compulsory schooling	56	1.41	0.97	-	2.04		53	1.63	1.17	-	2.27	
Vocational training	50	1					39	1				
Upper secondary education	58	1.64	1.34	-	2.02		34	0.82	0.67	-	1.01	
University education	70	1.98	1.51	-	2.60	<0.001	24	0.49	0.37	-	0.64	<0.001
**Civil status**												
Single, divorced, other	54	1					34	1				
Married	61	1.08	0.86	-	1.35	0.519	47	1.56	1.26	-	1.94	<0.001
**Children**												
No	53	1					34	1				
Yes	59	0.94	0.77	-	1.14	0.534	42	1.20	0.99	-	1.46	0.060
**BMI categories (kg/m2)**												
Underweight (<18)	71	2.18	1.26	-	3.79		40	1.21	0.74	-	1.98	
Normal weight (≥18/<25)	56	1					35	1				
Overweight (≥25/<30)	49	0.83	0.67	-	1.01		38	1.05	0.86	-	1.30	
Obese (≥30)	53	1.00	0.70	-	1.43	0.008	57	2.15	1.50	-	3.09	0.001
**Smoking**												
Current non-smoker	55	1					33	1				
Current smoker	55	1.02	0.86	-	1.21	0.838	45	1.74	1.46	-	2.07	<0.001

Abbreviations: BMI, Body Mass Index; CI, Confidence Interval; OR, Odds Ratio.

a calculated on weighted analysis (weights on: age, gender, language region, nationality).

b standardized proportions given in column percentages.

c global p-value calculated with a Wald test.

### Determinants of sporting activities

**Figure 3:**
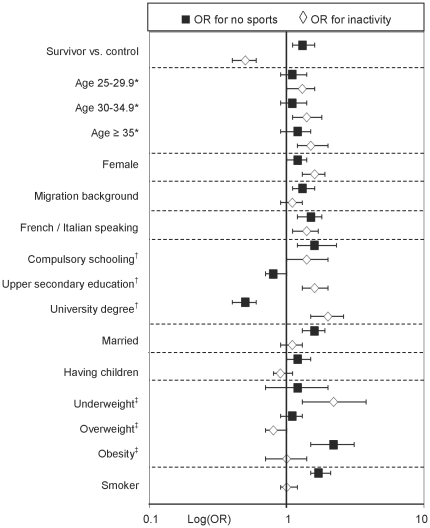
Association of socio-demographic risk factors with two types of physical activity: daily activities and sports. Effect sizes for two different outcomes assessing physical activity derived from two multivariable regression models with a combined dataset including childhood cancer survivors and healthy controls. Abbreviations: OR, Odds Ratio. * versus age <25 years. ^†^ versus vocational training. ^‡^ versus normal weight.

Determinants of sporting activities differed from determinants of daily activities (**Figure 3**). In the univariable model factors associated with lack of sports were: older age (OR for ≥35 years: 1.4, 95CI 1.1–1.8), female sex (OR 1.2, 95CI 1.0–1.4), migration background (OR 1.5, 95CI 1.3–1.8), French or Italian speaking (OR 1.4, 95CI 1.2–1.7), compulsory schooling only (OR 1.6, 95CI 1.2–2.2), being married (OR 1.7, 95CI 1.5–2.0), having children (OR 1.3, 95CI 1.1–1.5), being obese (OR 2.4, 95CI 1.7–3.3) and current smoking (OR 1.6, 95CI 1.4–1.9; **Table S2**). Participants with higher education participated more often in sports. Results from the multivariable model were similar to the univariable regression, only current age was no longer associated (**Table 2**). In contrast to daily activities, however, more survivors reported a lack of sporting activities (OR 1.3, 95CI 1.1–1.6). There was no evidence that the effect of risk factors differed between survivors and their peers (all p values for interaction >0.01).

### Association between clinical factors and physical activities in survivors

Adjusting for all socio-demographic and cultural factors listed in Table 2, none of the clinical factors (type of diagnosis, age at diagnosis, treatments or self-reported late effects) were significantly associated with daily activities (results from univariable models in **Table S3**). However, there was a trend for lower activity levels in survivors of neuroblastomas, soft tissue sarcomas, and germ cell tumors (**Table 3**). The same was true for clinical predictors of sporting activities (results from univariable models in **Table S3**): while there was no statistically significant association with clinical factors, there was a trend (p = 0.087) for reporting less sports in survivors of bone tumors (OR 2.0, 95CI 1.1–3.9), germ cell tumors (OR 2.2, 95CI 0.9–5.3), CNS tumors (OR 1.6, 95CI 0.9–3.0), neuroblastomas (OR 1.8, 95CI 0.8–4.0), and retinoblastomas (OR 1.8, 95CI 0.6–5.3; **Table 3**).

**Table 3:** Risk factors for inactivity and no sports in childhood cancer survivors from two multivariable regression models.

	Daily activities						Sports					
	Inactive	Multivariable regression					No sports	Multivariable regression				
	%^a^	OR	95% CI			p^b^	%^a^	OR	95% CI			p^b^
**Current age**												
0–24.9 years	43	1					36	1				
25–29.9 years	50	1.49	1.05	-	2.11		36	1.11	0.77	-	1.58	
30–34.9 years	54	1.46	0.93	-	2.29		43	1.19	0.76	-	1.87	
≥35 years	51	1.31	0.77	-	2.24	0.131	44	1.06	0.62	-	1.82	0.871
**Gender**												
Male	42	1					39	1				
Female	55	1.66	1.24	-	2.22	0.001	38	0.92	0.69	-	1.24	0.601
**Migration background**												
No	46	1					36	1				
Yes	53	1.18	0.84	-	1.64	0.336	45	1.34	0.96	-	1.85	0.084
**Language region**												
German speaking	44	1					37	1				
French/Italian speaking	61	1.87	1.33	-	2.63	<0.001	44	1.34	0.96	-	1.88	0.087
**Education**												
Compulsory schooling	42	1.87	1.07	-	3.27		51	1.72	1.01	-	2.93	
Vocational training	53	1					40	1				
Upper secondary education	52	1.43	1.05	-	1.95		36	0.85	0.62	-	1.17	
University education	61	1.83	1.03	-	3.25	0.017	26	0.51	0.27	-	0.97	0.016
**Civil status**												
Single, divorced, other	47	1					37	1				
Married	55	1.01	0.60	-	1.70	0.967	47	1.05	0.62	-	1.77	0.853
**Children**												
No	47	1					36	1				
Yes	58	1.40	0.79	-	2.48	0.248	51	1.79	1.01	-	3.17	0.045
**BMI categories (kg/m2)**												
Underweight (<18)	74	3.02	1.34	-	6.80		39	1.23	0.60	-	2.57	
Normal weight (≥18/<25)	48	1					36	1				
Overweight (≥25/<30)	43	1.01	0.71	-	1.45		38	0.99	0.69	-	1.42	
Obese (≥30)	54	1.46	0.81	-	2.63	0.037	59	2.28	1.27	-	4.10	0.044
**Smoking**												
Current non-smoker	48	1					35	1				
Current smoker	50	1.21	0.87	-	1.69	0.255	49	1.87	1.35	-	2.58	<0.001
**Age at diagnosis**												
0–4.9 years	43	1					38	1				
5–9.9 years	47	1.25	0.85	-	1.83		33	0.73	0.50	-	1.09	
≥10 years	52	1.42	0.96	-	2.10	0.205	42	1.01	0.69	-	1.50	0.167
**Treatment**												
Surgery only	41	0.81	0.42	-	1.54		35	0.66	0.35	-	1.27	
Chemotherapy^c^	48	1					37	1				
Radiotherapy^d^	50	0.86	0.60	-	1.22		40	0.99	0.70	-	1.42	
BMT	49	1.04	0.61	-	1.76	0.784	45	1.47	0.97	-	2.46	0.255
**Diagnosis (ICCC3 main groups)**												
Leukemias	46	1					37	1				
Lymphomas	50	1.06	0.71	-	1.58		35	0.87	0.58	-	1.31	
CNS tumors	40	0.91	0.48	-	1.72		43	1.59	0.86	-	2.96	
Neuroblastomas	55	1.64	0.74	-	3.63		45	1.82	0.82	-	4.03	
Retinoblastomas	50	1.44	0.47	-	4.36		44	1.76	0.59	-	5.26	
Renal & hepatic tumors^e^	43	1.02	0.55	-	1.89		24	0.66	0.33	-	1.32	
Bone tumors	58	1.34	0.69	-	2.59		56	2.01	1.05	-	3.87	
Soft tissue sarcomas	63	1.79	0.91	-	3.53		40	1.14	0.59	-	2.22	
Germ cell tumors	63	1.64	0.66	-	4.06		56	2.22	0.93	-	5.28	
Langerhans cell histiocytosis	36	0.69	0.30	-	1.55		32	0.87	0.38	-	1.95	
Other^f^	55	1.46	0.41	-	5.16	0.639	27	0.64	0.16	-	2.60	0.087
**Self-reported late effects**												
No late effects	45	1					36	1				
Somatic late effects	56	1.41	0.99	-	2.00		44	1.33	0.94	-	1.89	
Psychological late effects	48	0.93	0.57	-	1.51	0.116	40	1.02	0.63	-	1.66	0.230

Abbreviations: BMI, Body Mass Index; BMT, Bone Marrow Transplantation; Chemo, Chemotherapy; CI, Confidence Interval; CNS, Central Nervous System; ICCC-3, International Classification of Childhood Cancer Third Edition; OR, Odds Ratio; Radio, Radiotherapy.

a column percentages are given.

b global p-value calculated with a Wald test.

c chemotherapy may include surgery.

d radiotherapy may include chemotherapy or surgery.

e renal and hepatic tumors have been merged for this analysis.

f other malignant epithelial neoplasm, malignant melanomas and other or unspecified malignant neoplasm.

## Discussion

To our knowledge, this is the first population based study that compared different types of physical activities between childhood cancer survivors and healthy controls in Europe. Results differed between the two types of activities: while survivors reported more daily activities, they performed fewer sports than controls. Risk factors for inactivity did also differ between daily activities and sports. Demographic and cultural factors such as gender, age, education, BMI, smoking status and family status were the most important determinants of physical activity in both populations.

### Strengths and limitations

A major strength of our study is the population-based, representative and large sample of Swiss childhood cancer survivors. Thanks to the high response rate of 78%, selection bias plays a minor role. Demographic and medical information had been collected prospectively in the Swiss Childhood Cancer Registry. By weighting the analysis, we could compare proportions between the two populations which were standardized with respect to age, sex, nationality, and language region, allowing a valid comparison between survivors and controls. Finally, same questions had been used to assess outcomes and exposures in both studies.

There are some limitations to be acknowledged regarding the assessment of physical activities. First, a clear limitation, which this study shares with others [24], [25], [26], [27], [28] is, that physical activity was self-reported. Physical activity can only incompletely be assessed by questionnaire, and social desirability bias and subjective interpretation has to be considered. The current gold standard for assessment of physical activity is doubly labeled water [43], but the method is costly and rarely used. A second possibility is the use of accelerometers [43]. The questions on daily activities we used in our survey are internationally standardized and had been validated against accelerometer assessment in childhood cancer survivors with good correlations [15], [38]. The questions on sports have been used in the Swiss Health Survey since 1997 [36]. Second, a problem in the assessment of daily activities is, that the duration (minutes) of vigorous activities was not assessed in the control population, as it was assumed in the Swiss Health Survey that engagement in vigorous activities virtually always lasts more than 20 minutes. We used the same classification strategy for survivors and controls, neglecting the minutes of vigorous activities. This might have led to a slight overestimation of the daily activities but allows a fair comparison of the two populations. Third, we cannot fully separate daily activities and sports with the questions used. There might be some degree of overlapping. Despite this, there is evidence for clearly different risk profiles for daily activities and sports (**Figure 3**). Had we been able to make a clearer distinction between the two outcomes, the differences in risk factors would likely have been more extreme. Last, we cannot exclude that the different survey designs (written questionnaire in survivors, telephone interview in controls) might have influenced the results, with responders to the telephone survey being more prone to social desirability bias [44].

Finally, although it was a nationwide survey, the relative rarity of childhood cancer resulted in limited numbers of participants and thus limited power to show small differences between diagnostic groups.

### Comparison with other studies

Two studies from the US Childhood Cancer Survivor Study previously studied daily activities in a population-based sample of adult childhood cancer survivors, using similar questions and classifications [24], [25]. In contrast to our study, Ness et al. found that less survivors than controls engaged in sufficient daily activities. Among 9301 survivors of the Childhood Cancer Survivor Study compared to 2886 siblings, 45% survivors and 51% siblings reported sufficient daily activities [25]. This was slightly lower than among 2'648 adult survivors of ALL who participated in the Childhood Cancer Survivor Study compared to 110'623 controls from the Behavioral Risk Factor Surveillance System of the USA. In this group, 48% survivors and 52% controls reported sufficient daily activities [24].

These two studies also compared sporting activities, although assessed slightly different. Overall, the proportion engaging in sports was lower in the US studies than in our findings from Switzerland, both in survivors and controls. However, consistent with our findings fewer survivors than controls engaged in sports [24], [25]. In the study of Florin et al. 77% survivors reported no sports compared to 80% controls; Ness et al. reported 77% vs. 86%, respectively. Ness et al. found similar associations between sports and socio-demographic factors as we did in Switzerland (age, education, BMI, smoking), but also associations with certain diagnoses (medullablastoma and osteosarcoma) and treatments (cranial radiation or amputation).

Differences between our findings and those from the US might be explained by socio-demographic, clinical and cultural differences between Swiss and US survivors and controls, as well as differences in the information on diagnosis and treatment.

### Interpretation of the results

We found that risk factors for physical activity and comparisons between survivors and controls depend on the definition of the outcome, e.g. the way physical activity is assessed. Survivors reported more daily activities but fewer sports than their peers. In both survivors and controls, socio-demographic and cultural factors were important determinants of activity patterns, but they differed by type of activity (**Figure 3** **,** **Table 2**).

Previous studies found that higher education positively influences physical activity [45]. We confirmed this in our study for sporting activities. The inverse association we found between educational level and daily activities might be explained by more sedentary work and more working hours per day in higher educated persons.

In the analysis including childhood cancer survivors only, socio-demographic and cultural factors remained the main determinants of activity patterns, and clinical factors played a comparatively minor role. Only for the outcome “sports”, we found a disadvantage for survivors of certain types of cancers: bone tumors, germ cell tumors, retinoblastomas, neuroblastomas and CNS tumors. This might be due to cancer- or treatment related short- or long-term effects that impede participation in many sports, such as vision impairments after retinoblastoma, leg amputation after bone tumor or problems with balance or coordination after a CNS tumor.

In contrast, more survivors than controls reported sufficient daily activities. Perhaps survivors who cannot participate in targeted sports, due to performance limitations [46] or because of the “overprotected child syndrome” [47], they try to compensate this by including more activities into daily living. Alternatively it could be explained by the way daily activities are measured in the standardized instrument that we used, i.e. as “activities that make you breathe or sweat”. Survivors with physical handicaps, might tend to “breathe hard or sweat” more easily than peers during normal activities of daily living. A last potential explanation is selection bias: survivors in poor health might be both less likely to complete the questionnaire and to perform sports. These considerations might explain why the picture we gain from this survey contrasts with impressions from follow-up clinics, where survivors with the poorest outcomes are most likely to be seen.

### Implication for practice

Several intervention studies during or right after treatment of childhood cancer patients with promising results are mentioned in the literature [13], [14], [48]. However, only little is known on interventions that target enhancement of physical activity levels in adult survivors of childhood cancer many years after completion of treatment [14]. Therefore, our findings can inform daily practice and should feed into physical activity counseling during follow-up visits of current patients and long term survivors. Clinicians should keep in mind that several types of physical activity exist and risk profiles for inactivity differ depending on the type of activity. Depending on clinical factors (limitations for certain types of activities), individual preferences or risk profiles, different types of activities should be proposed to improve life-long engagement in physical activity.

For instance, survivors with certain performance-limiting late effects such as amputations or overweight that keep them from participating in certain sporting activities, could instead be motivated to include moderate physical activities into their daily living. From a health perspective, this type of moderate activity is sufficient to reduce the risk for chronic diseases [19], [22]. Thus, clinicians could give ideas on how to integrate activity into daily living: for example, climbing stairs instead of taking the elevator, riding a bicycle instead of taking a car, getting out of the bus a station earlier and walk the last meters, or balance on one leg while brushing teeth.

Importantly, the same socio-demographic and cultural factors determined activity patterns in survivors and their peers. This suggests that strategies to motivate childhood cancer survivors to be more active and structures to facilitate healthy activity behavior could be essentially similar as for the general population. Former patients can and should be integrated into physical activity interventions that aim for the general population, such as health-promoting school or work place interventions [49]. This does not exclude that high risk patients, such as those with multiple morbidities or those at high risk for cardiovascular, musculoskeletal, and neurocognitive late effects should be identified early and offered multiple health behavior counseling on an individual basis.

### Conclusion

Physical activity is a powerful tool to prevent chronic diseases and increases quality of life in healthy people [14], [50], [51]. Due to the vulnerability of cancer survivors and their high risk for chronic disease after treatment, a healthy lifestyle including regular physical activity is particularly important for this population [4], [16], [19], [52], [53]. Although we found that childhood cancer survivors were at least as active as their healthy peers, 48% did not reach recommended daily activity levels, 37% reported not doing any sports, and 21% reached neither recommendations for daily activities nor engaged in sports. These survivors are at a higher risk for developing late effects such as low bone mineral density, obesity, fatigue, cardiovascular diseases, low health related quality of life, or neurocognitive decline [9], [10], [11], [12], [15], [20]. As health behaviors tend to cluster together, inactive survivors might also be more prone to engage in other health compromising behaviors such as smoking, drinking or unhealthy diet [54]. This underlines the necessity to promote physical activity in childhood cancer survivors already early in follow-up. In addition, because risk factors for inactivity are essentially similar as in peers, survivors will benefit from all strategies that promote sports and daily physical activity in the general population.

## Supporting Information

Figure S1 **Questions on physical activity from the Swiss Childhood Cancer Survivor Study questionnaire (translated into English).** Same questions were asked to the controls in the Swiss Health Survey, excluding question 2 and 6.
(TIFF)

Table S1 **Characteristics of participants included in the analysis and non-participants^a^.** Abbreviations: ICCC-3, International Classification of Childhood Cancer Third Edition; CNS, Central Nervous System; SD, Standard Deviation. ^a^ non-participants included: survivors without current address (n = 199), who did not response (n = 252), who refused to participate (n = 79), who answered an abridged questionnaire (n = 98). ^b^ p-value calculated from chi-square statistics. ^c^ other malignant epithelial neoplasms, malignant melanomas and other or unspecified malignant neoplasms. ^d^ chemotherapy may include surgery. ^e^ radiotherapy may include chemotherapy or surgery. ^f^ p-value calculated from two-group mean-comparison test (t-test).
(DOCX)

Table S2 **Risk factors for inactivity and no sports from unadjusted regression modelsa (combined dataset including childhood cancer survivors and controls).** Abbreviations: BMI, Body Mass Index; CI, Confidence Interval; OR, Odds Ratio. ^a^ calculated on weighted analysis (weights on: age, gender, language region, nationality). ^b^ standardized proportions given in column percentages. ^c^ global p-value calculated with a Wald test.
(DOCX)

Table S3 **Risk factors for inactivity and no sports in childhood cancer survivors from unadjusted regression models.** Abbreviations: BMI, Body Mass Index; BMT, Bone Marrow Transplantation; Chemo, Chemotherapy; CI, Confidence Interval; CNS, Central Nervous System; ICCC-3, International Classification of Childhood Cancer Third Edition; OR, Odds Ratio; Radio, Radiotherapy. ^a^ column percentages are given. ^b^ global p-value calculated with a Wald test. ^c^ chemotherapy may include surgery. ^d^ radiotherapy may include chemotherapy or surgery. ^e^ renal and hepatic tumors have been merged for this analysis. ^f^ other malignant epithelial neoplasm, malignant melanomas and other or unspecified malignant neoplasm.
(DOCX)
