# Supplementary figures and images for: Daily Physical Activities and Sports in Adult Survivors of Childhood Cancer and Healthy Controls: A Population-Based Questionnaire Survey
Source: PLoS One. 2012 Apr 10;7(4):e34930. doi: 10.1371/journal.pone.0034930 (PMC3323587; doi:10.1371/journal.pone.0034930)

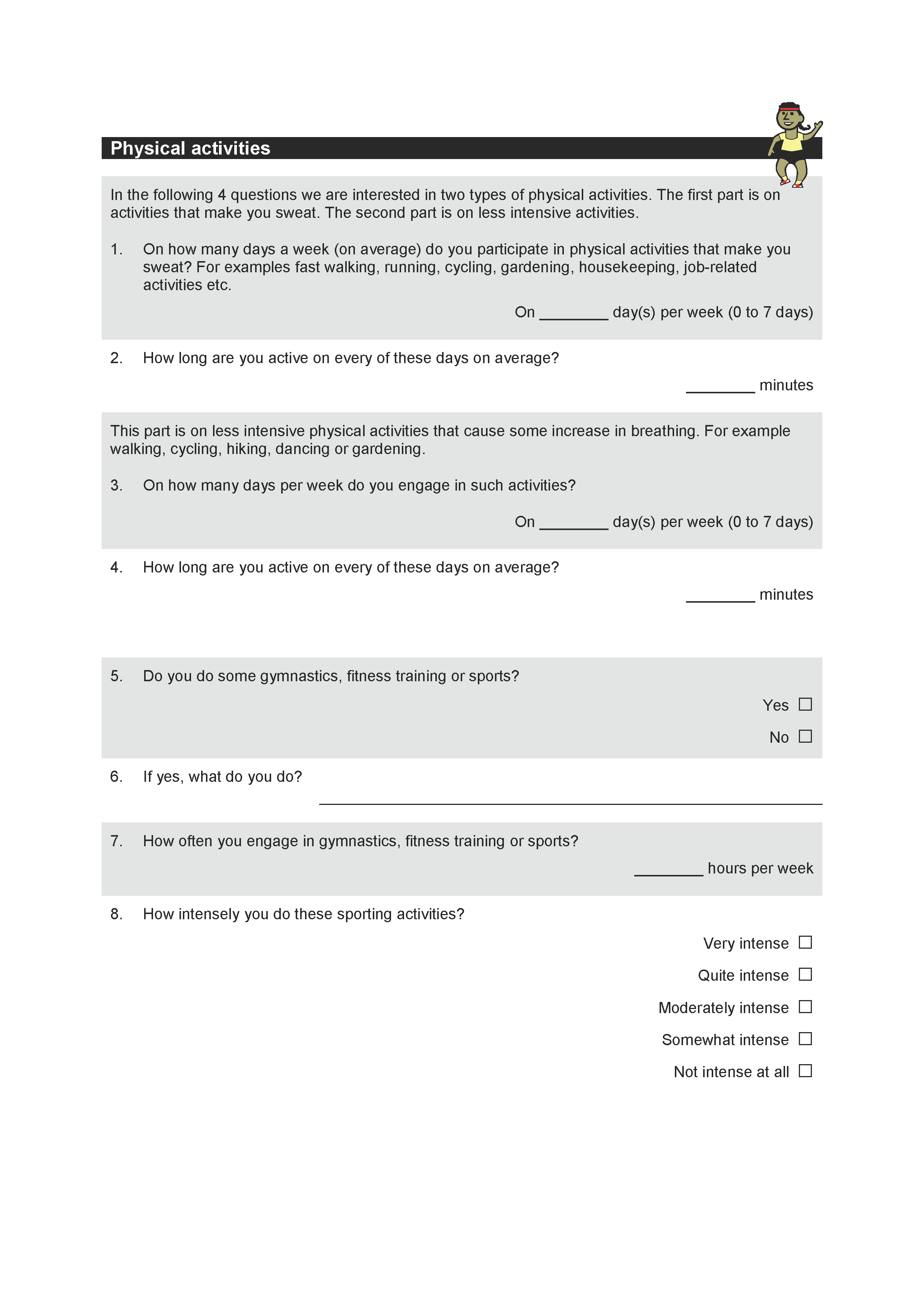

Supplement: Figure S1 — Questions on physical activity from the Swiss Childhood Cancer Survivor Study questionnaire (translated into English). Same questions were asked to the controls in the Swiss Health Survey, excluding question 2 and 6. (TIFF) [file pone.0034930.s001.tiff]
