# Supplementary material for: Daily Physical Activities and Sports in Adult Survivors of Childhood Cancer and Healthy Controls: A Population-Based Questionnaire Survey
Source: PLoS One. 2012 Apr 10;7(4):e34930. doi: 10.1371/journal.pone.0034930 (PMC3323587; doi:10.1371/journal.pone.0034930)
Supplement: Table S1 — Characteristics of participants included in the analysis and non-participantsa. Abbreviations: ICCC-3, International Classification of Childhood Cancer Third Edition; CNS, Central Nervous System; SD, Standard Deviation. a non-participants included: survivors without current address (n = 199), who did not response (n = 252), who refused to participate (n = 79), who answered an abridged questionnaire (n = 98). b p-value calculated from chi-square statistics. c other malignant epithelial neoplasms, malignant melanomas and other or unspecified malignant neoplasms. d chemotherapy may include surgery. e radiotherapy may include chemotherapy or surgery. f p-value calculated from two-group mean-comparison test (t-test). (DOCX) [file pone.0034930.s002.docx]

**Table S1. Characteristics of participants included in the analysis and non-participants^a^.**

|  | | **Participants** (n=1058) | |  | **Non participants** (n=628) | |  |
| --- | --- | --- | --- | --- | --- | --- | --- |
| **Characteristics** | | **n** | **%** |  | **n** | **%** | **p^b^** |
| **Gender** | |  |  |  |  |  |  |
|  | Male | 562 | 53 |  | 391 | 62 |  |
|  | Female | 496 | 47 |  | 237 | 38 | <0.001 |
| **Language** | |  |  |  |  |  |  |
|  | German | 815 | 77 |  | 423 | 67 |  |
|  | French | 214 | 20 |  | 183 | 29 |  |
|  | Italian | 29 | 3 |  | 22 | 4 | <0.001 |
| **Diagnosis (ICCC-3 main groups)** | | |  |  |  |  |  |
|  | Leukemias | 394 | 37 |  | 184 | 29 |  |
|  | Lymphomas | 219 | 21 |  | 153 | 25 |  |
|  | CNS tumors | 121 | 11 |  | 73 | 12 |  |
|  | Neuroblastomas | 36 | 3 |  | 29 | 5 |  |
|  | Retinoblastomas | 21 | 2 |  | 15 | 2 |  |
|  | Renal tumors | 60 | 6 |  | 27 | 4 |  |
|  | Hepatic tumors | 7 | 1 |  | 1 | 0 |  |
|  | Bone tumors | 54 | 5 |  | 24 | 4 |  |
|  | Soft tissue sarcomas | 58 | 6 |  | 42 | 7 |  |
|  | Germ cell tumors | 28 | 3 |  | 29 | 5 |  |
|  | Langerhans cell histiocytosis | 44 | 4 |  | 33 | 5 |  |
|  | Other^c^ | 13 | 1 |  | 14 | 2 | 0.008 |
| **Therapy** | |  |  |  |  |  |  |
|  | Surgery only | 95 | 9 |  | 66 | 11 |  |
|  | Chemotherapy^d^ | 489 | 47 |  | 271 | 44 |  |
|  | Radiotherapy^e^ | 378 | 36 |  | 260 | 42 |  |
|  | Bone marrow transplantation | 87 | 8 |  | 20 | 3 | <0.001 |
|  |  | **mean** | **SD** |  | **mean** | **SD** | **p^f^** |
| Age at survey | | 28.1 | 6.2 |  | 28.4 | 6.5 | 0.281 |
| Age at diagnosis | | 8.3 | 4.8 |  | 8.1 | 4.7 | 0.417 |
| Time since diagnosis | | 19.8 | 6.6 |  | 20.3 | 7.1 | 0.117 |
