# Supplementary material for: Daily Physical Activities and Sports in Adult Survivors of Childhood Cancer and Healthy Controls: A Population-Based Questionnaire Survey
Source: PLoS One. 2012 Apr 10;7(4):e34930. doi: 10.1371/journal.pone.0034930 (PMC3323587; doi:10.1371/journal.pone.0034930)
Supplement: Table S2 — Risk factors for inactivity and no sports from unadjusted regression modelsa (combined dataset including childhood cancer survivors and controls). Abbreviations: BMI, Body Mass Index; CI, Confidence Interval; OR, Odds Ratio. a calculated on weighted analysis (weights on: age, gender, language region, nationality). b standardized proportions given in column percentages. c global p-value calculated with a Wald test. (DOCX) [file pone.0034930.s003.docx]

Table S2. Risk factors for inactivity and no sports from unadjusted regression models^a^ (combined dataset including childhood cancer survivors and controls).

|  | | **Daily activities** | | | | | | |  | **Sports** | | | | | | |
| --- | --- | --- | --- | --- | --- | --- | --- | --- | --- | --- | --- | --- | --- | --- | --- | --- |
|  | | **Inactive** |  | **Univariable regression** | | | | |  | **No sports** |  | **Univariable regression** | | | | |
|  |  | **%**^b^ |  | **OR** | **95% CI** | | | **p**^c^ |  | **%**^b^ |  | **OR** | **95% CI** | | | **p**^c^ |
| **Population** | |  |  |  |  |  |  |  |  |  |  |  |  |  |  |  |
|  | Controls | 63 |  | 1 |  |  |  |  |  | 35 |  | 1 |  |  |  |  |
|  | Survivors | 48 |  | 0.55 | 0.47 | - | 0.63 | <0.001 |  | 38 |  | 1.15 | 0.99 | - | 1.32 | 0.067 |
| **Current age** | |  |  |  |  |  |  |  |  |  |  |  |  |  |  |  |
|  | 0-24.9 years | 50 |  | 1 |  |  |  |  |  | 34 |  | 1 |  |  |  |  |
|  | 25-29.9 years | 58 |  | 1.36 | 1.13 | - | 1.63 |  |  | 35 |  | 1.04 | 0.87 | - | 1.26 |  |
|  | 30-34.9 years | 58 |  | 1.38 | 1.13 | - | 1.70 |  |  | 40 |  | 1.24 | 1.01 | - | 1.53 |  |
|  | ≥ 35 years | 60 |  | 1.47 | 1.17 | - | 1.85 | <0.001 |  | 43 |  | 1.41 | 1.12 | - | 1.76 | 0.009 |
| **Gender** | |  |  |  |  |  |  |  |  |  |  |  |  |  |  |  |
|  | Male | 50 |  | 1 |  |  |  |  |  | 35 |  | 1 |  |  |  |  |
|  | Female | 61 |  | 1.58 | 1.36 | - | 1.84 | <0.001 |  | 39 |  | 1.17 | 1.01 | - | 1.36 | 0.039 |
| **Migration background** | |  |  |  |  |  |  |  |  |  |  |  |  |  |  |  |
|  | No | 54 |  | 1 |  |  |  |  |  | 34 |  | 1 |  |  |  |  |
|  | Yes | 57 |  | 1.11 | 0.94 | - | 1.32 | 0.231 |  | 44 |  | 1.52 | 1.29 | - | 1.79 | <0.001 |
| **Language region** | |  |  |  |  |  |  |  |  |  |  |  |  |  |  |  |
|  | German speaking | 53 |  | 1 |  |  |  |  |  | 35 |  | 1 |  |  |  |  |
|  | French / Italian speaking | 61 |  | 1.37 | 1.16 | - | 1.63 | <0.001 |  | 43 |  | 1.40 | 1.18 | - | 1.65 | <0.001 |
| **Education** | |  |  |  |  |  |  |  |  |  |  |  |  |  |  |  |
|  | Compulsory schooling | 56 |  | 1.27 | 0.90 | - | 1.78 |  |  | 52 |  | 1.64 | 1.21 | - | 2.23 |  |
|  | Vocational training | 50 |  | 1 |  |  |  |  |  | 39 |  | 1 |  |  |  |  |
|  | Upper secondary education | 58 |  | 1.42 | 1.18 | - | 1.72 |  |  | 34 |  | 0.79 | 0.65 | - | 0.96 |  |
|  | University education | 70 |  | 2.32 | 1.79 | - | 3.01 | <0.001 |  | 25 |  | 0.50 | 0.39 | - | 0.65 | <0.001 |
| **Civil status** | |  |  |  |  |  |  |  |  |  |  |  |  |  |  |  |
|  | Single, divorced, other | 54 |  | 1 |  |  |  |  |  | 34 |  | 1 |  |  |  |  |
|  | Married | 61 |  | 1.34 | 1.13 | - | 1.58 | 0.001 |  | 47 |  | 1.74 | 1.48 | - | 2.04 | <0.001 |
| **Children** | |  |  |  |  |  |  |  |  |  |  |  |  |  |  |  |
|  | No | 53 |  | 1 |  |  |  |  |  | 35 |  | 1 |  |  |  |  |
|  | Yes | 59 |  | 1.26 | 1.08 | - | 1.47 | 0.002 |  | 41 |  | 1.28 | 1.11 | - | 1.48 | 0.001 |
| **BMI categories (kg/m2)** | |  |  |  |  |  |  |  |  |  |  |  |  |  |  |  |
|  | Underweight (<18) | 71 |  | 1.91 | 1.17 | - | 3.10 |  |  | 40 |  | 1.29 | 0.83 | - | 2.00 |  |
|  | Normal weight (≥18/<25) | 56 |  | 1 |  |  |  |  |  | 34 |  | 1 |  |  |  |  |
|  | Overweight (≥25/<30) | 49 |  | 0.74 | 0.61 | - | 0.89 |  |  | 37 |  | 1.15 | 0.95 | - | 1.39 |  |
|  | Obese (≥30) | 53 |  | 0.88 | 0.64 | - | 1.24 | <0.001 |  | 56 |  | 2.42 | 1.74 | - | 3.36 | <0.001 |
| **Smoking** | |  |  |  |  |  |  |  |  |  |  |  |  |  |  |  |
|  | Current non-smoker | 55 |  | 1 |  |  |  |  |  | 33 |  | 1 |  |  |  |  |
|  | Current smoker | 55 |  | 1.01 | 0.87 | - | 1.19 | 0.862 |  | 45 |  | 1.64 | 1.40 | - | 1.91 | <0.001 |
