# Supplementary material for: Daily Physical Activities and Sports in Adult Survivors of Childhood Cancer and Healthy Controls: A Population-Based Questionnaire Survey
Source: PLoS One. 2012 Apr 10;7(4):e34930. doi: 10.1371/journal.pone.0034930 (PMC3323587; doi:10.1371/journal.pone.0034930)
Supplement: Table S3 — Risk factors for inactivity and no sports in childhood cancer survivors from unadjusted regression models. Abbreviations: BMI, Body Mass Index; BMT, Bone Marrow Transplantation; Chemo, Chemotherapy; CI, Confidence Interval; CNS, Central Nervous System; ICCC-3, International Classification of Childhood Cancer Third Edition; OR, Odds Ratio; Radio, Radiotherapy. a column percentages are given. b global p-value calculated with a Wald test. c chemotherapy may include surgery. d radiotherapy may include chemotherapy or surgery. e renal and hepatic tumors have been merged for this analysis. f other malignant epithelial neoplasm, malignant melanomas and other or unspecified malignant neoplasm. (DOCX) [file pone.0034930.s004.docx]

Table S3. Risk factors for inactivity and no sports in childhood cancer survivors from unadjusted regression models.

|  | | **Daily activities** | | | | | | | |  | **Sports** | | | | | | |
| --- | --- | --- | --- | --- | --- | --- | --- | --- | --- | --- | --- | --- | --- | --- | --- | --- | --- |
|  | | **Inactive** | |  | **Univariable regression** | | | | |  | **No sports** |  | **Univariable regression** | | | | |
|  |  | **%**^a^ | |  | **OR** | **95% CI** | | | **p**^b^ |  | **%**^a^ |  | **OR** | **95% CI** | | | **p**^b^ |
| **Current age** | |  | |  |  |  |  |  |  |  |  |  |  |  |  |  |  |
|  | 0-24.9 years | 42 | |  | 1 |  |  |  |  |  | 37 |  | 1 |  |  |  |  |
|  | 25-29.9 years | 50 | |  | 1.38 | 1.01 | - | 1.87 |  |  | 36 |  | 0.98 | 0.72 | - | 1.34 |  |
|  | 30-34.9 years | 52 | |  | 1.47 | 1.03 | - | 2.09 |  |  | 41 |  | 1.21 | 0.85 | - | 1.73 |  |
|  | ≥ 35 years | 53 | |  | 1.53 | 1.03 | - | 2.67 | <0.001 |  | 44 |  | 1.37 | 0.93 | - | 2.01 | 0.295 |
| **Gender** | |  | |  |  |  |  |  |  |  |  |  |  |  |  |  |  |
|  | Male | 42 | |  | 1 |  |  |  |  |  | 38 |  | 1 |  |  |  |  |
|  | Female | 54 | |  | 1.63 | 1.27 | - | 2.09 | <0.001 |  | 39 |  | 1.03 | 0.80 | - | 1.32 | 0.827 |
| **Migration background** | |  | |  |  |  |  |  |  |  |  |  |  |  |  |  |  |
|  | No | 47 | |  | 1 |  |  |  |  |  | 36 |  | 1 |  |  |  |  |
|  | Yes | 52 | |  | 1.24 | 0.92 | - | 1.65 | 0.152 |  | 44 |  | 1.39 | 1.04 | - | 1.85 | 0.026 |
| **Language region** | |  | |  |  |  |  |  |  |  |  |  |  |  |  |  |  |
|  | German speaking | 44 | |  | 1 |  |  |  |  |  | 37 |  | 1 |  |  |  |  |
|  | French / Italian speaking | 60 | |  | 1.91 | 1.42 | - | 2.58 | <0.001 |  | 43 |  | 1.31 | 0.98 | - | 1.76 | 0.071 |
| **Education** | |  | |  |  |  |  |  |  |  |  |  |  |  |  |  |  |
|  | Compulsory schooling | 55 | |  | 1.72 | 1.06 | - | 2.81 |  |  | 48 |  | 1.39 | 0.88 | - | 2.20 |  |
|  | Vocational training | 42 | |  | 1 |  |  |  |  |  | 40 |  | 1 |  |  |  |  |
|  | Upper secondary education | 52 | |  | 1.53 | 1.16 | - | 2.01 |  |  | 35 |  | 0.81 | 0.61 | - | 1.08 |  |
|  | University education | 60 | |  | 2.09 | 1.27 | - | 3.44 | <0.001 |  | 26 |  | 0.51 | 0.30 | - | 0.89 | 0.013 |
| **Civil status** | |  | |  |  |  |  |  |  |  |  |  |  |  |  |  |  |
|  | Single, divorced, other | 46 | |  | 1 |  |  |  |  |  | 37 |  | 1 |  |  |  |  |
|  | Married | 56 | |  | 1.45 | 1.04 | - | 2.02 | 0.028 |  | 46 |  | 1.47 | 1.06 | - | 2.04 | 0.020 |
| **Children** | |  | |  |  |  |  |  |  |  |  |  |  |  |  |  |  |
|  | No | 46 | |  | 1 |  |  |  |  |  | 36 |  | 1 |  |  |  |  |
|  | Yes | 57 | |  | 1.51 | 1.05 | - | 2.18 | 0.028 |  | 50 |  | 1.74 | 1.21 | - | 2.50 | 0.003 |
| **BMI categories (kg/m2)** | |  | |  |  |  |  |  |  |  |  |  |  |  |  |  |  |
|  | Underweight (<18) | 72 | |  | 2.92 | 1.39 | - | 6.15 |  |  | 41 |  | 1.21 | 0.62 | - | 2.38 |  |
|  | Normal weight (≥18/<25) | 47 | |  | 1 |  |  |  |  |  | 36 |  | 1 |  |  |  |  |
|  | Overweight (≥25/<30) | 42 | |  | 0.82 | 0.60 | - | 1.13 |  |  | 38 |  | 1.07 | 0.78 | - | 1.47 |  |
|  | Obese (≥30) | 53 | |  | 1.28 | 0.76 | - | 2.15 | <0.001 |  | 57 |  | 2.35 | 1.41 | - | 3.94 | 0.013 |
| **Smoking** | |  | |  |  |  |  |  |  |  |  |  |  |  |  |  |  |
|  | Current non-smoker | 47 | |  | 1 |  |  |  |  |  | 35 |  | 1 |  |  |  |  |
|  | Current smoker | 48 | |  | 1.04 | 0.77 | - | 1.39 | 0.814 |  | 49 |  | 1.80 | 1.35 | - | 2.40 | <0.001 |
| **Age at diagnosis** | |  | |  |  |  |  |  |  |  |  |  |  |  |  |  |  |
|  | 0-4.9 years | 43 | |  | 1 |  |  |  |  |  | 38 |  | 1 |  |  |  |  |
|  | 5-9.9 years | 47 | |  | 1.19 | 0.86 | - | 1.65 |  |  | 34 |  | 0.83 | 0.60 | - | 1.17 |  |
|  | ≥ 10 years | 51 | |  | 1.37 | 1.02 | - | 1.84 | 0.108 |  | 41 |  | 1.12 | 0.83 | - | 1.50 | 0.204 |
| **Treatment** | |  | |  |  |  |  |  |  |  |  |  |  |  |  |  |  |
|  | Surgery only | 42 | |  | 0.77 | 0.49 | - | 1.20 |  |  | 35 |  | 0.92 | 0.58 | - | 1.45 |  |
|  | Chemotherapy^c^ | 48 | |  | 1 |  |  |  |  |  | 37 |  | 1 |  |  |  |  |
|  | Radiotherapy^d^ | 50 | |  | 1.06 | 0.80 | - | 1.39 |  |  | 40 |  | 1.15 | 0.87 | - | 1.52 |  |
|  | BMT | 47 | |  | 0.95 | 0.60 | - | 1.52 | 0.592 |  | 47 |  | 1.54 | 0.97 | - | 2.45 | 0.241 |
| **Diagnosis (ICCC3 main groups)** | |  |  |  |  |  |  |  |  |  |  |  |  |  |  |  |  |
|  | Leukemias | 45 | |  | 1 |  |  |  |  |  | 39 |  | 1 |  |  |  |  |
|  | Lymphomas | 50 | |  | 1.22 | 0.87 | - | 1.72 |  |  | 35 |  | 0.86 | 0.61 | - | 1.22 |  |
|  | CNS tumors | 42 | |  | 0.86 | 0.56 | - | 1.33 |  |  | 41 |  | 1.08 | 0.71 | - | 1.65 |  |
|  | Neuroblastomas | 51 | |  | 1.28 | 0.64 | - | 2.56 |  |  | 43 |  | 1.19 | 0.59 | - | 2.40 |  |
|  | Retinoblastomas | 57 | |  | 1.61 | 0.66 | - | 3.92 |  |  | 43 |  | 1.19 | 0.49 | - | 2.90 |  |
|  | Renal & hepatic tumors^e^ | 45 | |  | 0.98 | 0.57 | - | 1.66 |  |  | 27 |  | 0.58 | 0.33 | - | 1.04 |  |
|  | Bone tumors | 57 | |  | 1.60 | 0.88 | - | 2.88 |  |  | 54 |  | 1.86 | 1.04 | - | 3.32 |  |
|  | Soft tissue sarcomas | 57 | |  | 1.61 | 0.92 | - | 2.84 |  |  | 37 |  | 0.93 | 0.52 | - | 1.65 |  |
|  | Germ cell tumors | 67 | |  | 2.42 | 1.06 | - | 5.53 |  |  | 57 |  | 2.12 | 0.98 | - | 4.61 |  |
|  | Langerhans cell histiocytosis | 40 | |  | 0.81 | 0.41 | - | 1.57 |  |  | 33 |  | 0.77 | 0.39 | - | 1.50 |  |
|  | Other^f^ | 46 | |  | 1.04 | 0.34 | - | 3.15 | 0.259 |  | 23 |  | 0.48 | 0.13 | - | 1.76 | 0.079 |
| **Self-reported late effects** | |  | |  |  |  |  |  |  |  |  |  |  |  |  |  |  |
|  | No late effects | 45 | |  | 1 |  |  |  |  |  | 36 |  | 1 |  |  |  |  |
|  | Somatic late effects | 57 | |  | 1.61 | 1.19 | - | 2.16 |  |  | 45 |  | 1.46 | 1.08 | - | 1.96 |  |
|  | Psychological late effects | 47 | |  | 1.07 | 0.69 | - | 1.64 | 0.008 |  | 39 |  | 1.11 | 0.72 | - | 1.70 | 0.044 |
